# Neurosensory symptoms and muscle cramping in morphea: A cross‐sectional study and review

**DOI:** 10.1002/ski2.453

**Published:** 2024-12-01

**Authors:** Amanda Christine F. Esquivel, Christopher B. Hansen

**Affiliations:** ^1^ Department of Dermatology University of the Philippines Philippine General Hospital Manilla Philippines; ^2^ Department of Dermatology University of Utah Salt Lake City Utah USA

Dear Editor,

Morphea is a rare chronic fibrosing disorder of the skin and underlying tissues, with an estimated incidence of 2.7 per 100 000 individuals. It affects both children and adults, but affects females more than males.^1^ Clinical manifestations primarily include inflammatory patches or plaques that transition into sclerotic, then eventually atrophic, plaques and nodules. These lesions are often described as being asymptomatic. When the neurosensory symptoms are described, itching and pain are occasionally mentioned, though they are usually reported as occurring prior to the onset of the lesions.^2^, ^3^ Other neurosensory complaints such as burning, tingling, and muscle cramping are not commonly discussed. Neurosensory symptoms are likely underreported in morphea and frequently go unasked in practice, leading to possible gaps in patient care. We aim to describe the frequency of these different neurosensory symptoms in our morphea clinic.

This was a cross‐sectional retrospective review of morphea in a cohort of adults and children seen by the same provider at the University of Utah, Department of Dermatology, from January 2015–April 2024. The study included patients of all ages who were determined to have morphea based on clinical and/or histologic features.

We collected patient demographics, subtype of morphea based on the Laxer and Zulian classification^4^ (plaque/circumscribed, linear, generalised, pansclerotic, mixed, or other), and the presence or absence of the following neurosensory complaints (itch or pruritus, pain, burning or tingling, dysaesthesia, and muscle cramps), from their initial visit and based on spontaneous reporting by the patients.

We identified 119 patients from our cohort who met the criteria. 102 (85.7%) patients were female. Mean age of diagnosis was 35.6. Frequencies of morphea subtypes were as follows: plaque (*n* = 49, 41.2%), linear (*n* = 35, 29.4%), generalised (*n* = 25, 21.0%), pansclerotic (*n* = 1, 0.8%), and mixed (*n* = 6, 5.0%). Three patients with nodular morphea (*n* = 3, 2.5%) did not fit into those subtypes.

Although 37 (31.09%) patients reported no symptoms at all, nearly half, 55 (46.2%), patients reported itch or pruritus; 36 (30.3%) reported pain; 21 (17.7%) reported burning or tingling; 8 (6.7%) reported muscle cramps; and 25 (21.0%) reported dysaesthesia, or any abnormal sensation—usually “tightening” or “numbness”—that did not fit into the other categories. This data is summarised in Table 1.

**TABLE 1:** Summary of morphea subtypes and frequency of reported neurosensory symptoms.

	Gender	Age of visit	Itch or pruritus	Pain	Burning or tingling	Muscle cramps	Dysaesthesia	No reported symptoms
Plaque or circumscribed (*N* = 49)	*M* = 12	33.3 (5–80)	24 (49.0)	7 (14.3)	6 (12.2)	2 (4.1)	3 (6.1)	21 (42.9)
	*F* = 37							
Linear (*N* = 35)	*M* = 2	28.8 (7–55)	13 (37.1)	15 (42.9)	7 (20.0)	4 (11.4)	8 (22.9)	8 (22.9)
	*F* = 33							
Generalised (*N* = 25)	*M* = 2	48.8 (5–79)	14 (56.0)	10 (40.0)	7 (28.0)	1 (4.0)	13 (52.0)	4 (16)
	*F* = 23							
Pansclerotic (*N* = 1)	*M* = 0	58 (58)	1 (100)	1 (100)	1 (100)	0	0	0
	*F* = 1							
Mixed (*N* = 6)	*M* = 1	34.3 (13–58)	2 (33.3)	2 (33.3)	0	1 (16.7)	1 (16.7)	3 (50.0)
	*F* = 5							
Nodular (*N* = 3)	*M* = 0	38 (25–63)	1 (33.3)	1 (33.3)	0	0	0	1 (33.3)
	*F* = 3							

When we look at morphea subtypes and the neurosensory symptoms reported by patients of each subtype, we see that across subtypes, at least half of patients reported at least one of the neurosensory symptoms of itch, pain, burning or tingling, cramping, or dysaesthesia.

Studies looking at the neurologic involvement in morphea generally focus on central nervous system manifestations, such as headaches, seizures, and focal neurologic deficits.^5^ To our knowledge, there are no studies specifically describing the frequency of neurosensory symptoms of itch, pain, tingling or burning, or muscle cramping in morphea patients, despite the fact that these symptoms can play a substantial role in the quality of life of patients.

Pathophysiology of morphea is still not completely understood, but there is evidence supporting the role of autoimmune dysregulation with abnormal cytokine production and vascular dysfunction in the disease. Environmental factors, such as radiation, skin trauma, infections, or environmental exposures that trigger a cascade of T‐cell driven inflammation in predisposed individuals. The resulting endothelial damage stimulates the release of cytokines that upregulate the expression of adhesion molecules, including vascular cell adhesion molecule‐1, intercellular adhesion molecule‐1 and E‐cadherin. These, in turn, will recruit pro‐inflammatory TH1 and TH17 cells and associated profibrotic cytokines such as interleukin (IL) 4, IL 6 and transforming growth factor beta, which are capable of stimulating fibroblast production of collagen and other extracellular matrix proteins.^6^, ^7^

The pathophysiology of the neurosensory symptoms in morphea is yet to be explained. Some have mentioned that the pruritus in morphea could be associated to xerosis,^3^ but that does not explain the other symptoms. Our proposed mechanism would be that the same process involved in autoimmune dysregulation can also inflame nearby nerves, resulting in pain, burning, or tingling. Another possible speculation would be that the excess collagen deposition and progressive sclerosis which leads to vascular dysfunction also eventually leads to small nerve entrapment and disruption/dysfunction.

Also of interest in our cohort are the eight patients who complained of muscle cramping, which is not often reported in the literature. Of those patients, three had linear morphea, two had plaque or circumscribed morphea, one had generalised morphea, and one had mixed (generalised and linear) morphea. The cramping was always reported in the area of morphea involvement: two patients who had morphea on the upper and lower extremities reported cramping corresponding to those areas in the upper and lower extremities, four patients reported cramping in the lower extremities, and two patients reported cramping in the head and neck. Specific muscles involved were not identified on chart review. There have been a few case reports of morphea patients who complain of muscle spasms or muscle cramping, but it is usually reported in the linear and generalised types, which can extend deeper to affect muscle, tendon, and even bone. It is unusual in the plaque type. Further studies are needed in order to elucidate this phenomenon.

It has been reported that the symptoms of itch and pain can be stressors affecting health‐related quality of life in patients with morphea.^8^ These neurosensory symptoms might actually be overlooked by the healthcare team, especially because morphea is generally thought of as being asymptomatic. In contrast, only 31.1% of our morphea cohort reported being asymptomatic; the majority reported itch, pain, burning, tingling, or cramping when probed. The relatively high frequency of these neurosensory symptoms— nearly half (46.22%) of our cohort complained of itch or pruritus, and more than a quarter (30.25%) complained of pain—reflects the need for healthcare professionals to probe about these symptoms, so that prompt and adequate treatment may be provided.

Though there is no definitive treatment for these neurosensory symptoms related to morphea, in our experience, anti‐inflammatory medications, such as topical steroids and calcineurin inhibitors, systemic immunosuppressants, and medications for neuropathic pain, such as gabapentin and pregabalin, may have some benefit, though more studies are needed to see what could best serve this subset of patients.

We looked at a relatively large sample of morphea patients seen over a 9‐year period. Further study directions that could be undertaken could correlate the presence of these neurosensory symptoms to morphea disease activity and disease stage. This study was limited by its retrospective nature, possible inconsistent reporting of clinical information, and single‐centre implementation, which can lead to incomplete data and selection bias.

In conclusion, we describe the neurosensory symptoms in our adult and children morphea cohort and emphasise that these symptoms occur more frequently than typically reported in the literature. It is important for clinicians to ask about, and recognise, the presence and frequency of these symptoms, in both active and inactive disease, so that patients are adequately treated.

## CONFLICT OF INTEREST STATEMENT

The authors declare no conflicts of interest.

## AUTHOR CONTRIBUTIONS

**Amanda Christine F. Esquivel**: Conceptualisation (equal); Data curation (equal); Formal analysis (equal); Visualisation (equal); Writing—original draft (lead); Writing—review & editing (supporting). **Christopher B. Hansen**: Conceptualisation (equal); Data curation (equal); Formal analysis (equal); Investigation (equal); Methodology (equal); Supervision (lead); Visualisation (equal); Writing—original draft (supporting); Writing—review & editing (lead).

## FUNDING INFORMATION

This article received no specific grant from any funding agency in the public, commercial, or not‐for‐profit sectors.

## ETHICS STATEMENT

This study has been approved by the Institutional Review Board of the University of Utah, with reference number: IRB 00076927.

## PATIENT CONSENT

Not applicable.

## Data Availability

The data that support the findings of this study are in this article.
